# Enhancing motor imagery practice using synchronous action observation

**DOI:** 10.1007/s00426-022-01768-7

**Published:** 2022-12-27

**Authors:** Daniel L. Eaves, Nicola J. Hodges, Gavin Buckingham, Giovanni Buccino, Stefan Vogt

**Affiliations:** 1 School of Biomedical, Nutritional and Sport Sciences, Newcastle University, Newcastle upon Tyne, UK; 2 School of Kinesiology, University of British Columbia, Vancouver, Canada; 3 Department of Sport and Health Sciences, College of Life and Environmental Sciences, University of Exeter, Exeter, UK; 4 Division of Neuroscience, IRCCS San Raffaele and Vita Salute San Raffaele University, Milan, Italy; 5 Department of Psychology, Lancaster University, Lancaster, UK

## Abstract

In this paper, we discuss a variety of ways in which practising motor actions by means of motor imagery (MI) can be enhanced via synchronous action observation (AO), that is, by AO + MI. We review the available research on the (mostly facilitatory) behavioural effects of AO + MI practice in the *early stages of skill acquisition*, discuss possible theoretical explanations, and consider several issues related to the choice and presentation schedules of suitable models. We then discuss considerations related to AO + MI practice at *advanced skill levels*, including expertise effects, practical recommendations such as focussing attention on specific aspects of the observed action, using just-ahead models, and possible effects of the perspective in which the observed action is presented. In section “Coordinative AO + MI”, we consider scenarios where the observer imagines performing an action that complements or responds to the observed action, as a promising and yet under-researched application of AO + MI training. In section “The dual action simulation hypothesis of AO + MI”, we review the neurocognitive hypothesis that AO + MI practice involves two parallel action simulations, and we consider opportunities for future research based on recent neuroimaging work on parallel motor representations. In section “AO + MI training in motor rehabilitation”, we review applications of AO, MI, and AO + MI training in the field of neurorehabilitation. Taken together, this evidence-based, exploratory review opens a variety of avenues for future research and applications of AO + MI practice, highlighting several clear advantages over the approaches of purely AO- or MI-based practice.

## Introduction

In this paper, we explore a variety of ways in which action observation (AO) can interact with and inform motor imagery (MI). This interaction can take two basic forms: *asynchronous* interaction between AO and MI, and *synchronous* AO and MI. In the former case, AO would happen at a different time to MI, such as first observing an action and later engaging in MI, or when systematically alternating between AO and MI. In synchronous AO and MI (or AO + MI for short), a person would observe an action and *at the same time* imagine performing either the same or a different, coordinated action (see Glossary of Terms). The first, asynchronous scenario can be seen as a subset of observational learning, where several reviews of the related literature already exist (e.g. Hodges, 2017; Hodges et al., 2007; McCullagh et al., 2012; Ramsey et al., 2021; Rizzolatti et al., 2021). Although we are principally interested in both forms of combined AO and MI, in this paper, we focus on the second scenario of synchronous AO + MI. This decision is due to the uniqueness of this form of practice from either observational learning or MI practice (for reviews on MI practice, see Ladda et al., 2021; Schuster et al., 2011; Simonsmeier et al., 2021; Toth et al., 2020), and due to the currently strong research interest in this type of practice.

Before publication of our related position papers (Eaves et al., 2016a; Vogt et al., 2013), only a handful of studies was available on the topic of synchronous AO + MI. Previously, AO and MI were seen (and instructed) as two separate forms of covert re-enactment, and they were predominantly studied by different research groups with little cross-referencing. Over the past decade, however, it is encouraging to see the research interest into AO + MI has gathered significant momentum. Most of the available studies have focussed on the *immediate* effects of synchronous AO + MI instructions on neurophysiological and behavioural parameters (for reviews, see Eaves et al., 2016a; Emerson et al., 2018; McNeill et al., 2020; Vogt et al., 2013; Wright et al., 2021). In the current paper, our focus is instead on the opportunities of synchronous AO + MI as a form of *practising* motor actions. Akin to the research that has explored the immediate effects of AO + MI, the existing training studies broadly demonstrate facilitatory effects for AO + MI practice compared to the separate protocols of either AO or MI (see section “Empirical evidence”). We consider the implications of these results for current theory and future research.

On a theoretical level, it remains unclear in what ways MI, which is generally accepted as *the* prototypical form of motor simulation (Jeannerod, 1994, 2001), might interact with and be informed by synchronous AO. Furthermore, a dominant role for MI over concurrent AO was highlighted in two recent studies, which assessed corticospinal excitability during AO + MI of rhythmical finger movements (Bruton et al., 2020; Meers et al., 2020, see section “The dual action simulation hypothesis of AO + MI”). One might, therefore, wonder, both on theoretical and empirical grounds, what the combination of AO and MI brings to the table that MI alone cannot deliver, or might deliver less efficiently. Indeed, engaging in synchronous AO + MI can be more demanding in terms of the neurocognitive resources than engaging in either pure AO or pure MI (e.g. Eaves et al., 2016b; Emerson et al., 2022), and setting up an AO + MI intervention can be rather time-consuming to produce appropriate videos and to schedule their presentation. Thus, *why should one even bother with AO* + *MI interventions when MI is such an elegant alternative*?^1^.

To address this core question, our paper is organised as follows: in section “AO + MI training in the early stages of skill acquisition”, we focus on the range of possible contributions of AO to MI training in the early stages of skill acquisition, highlighting several novel areas for future research. In section “AO + MI training at advanced skill levels”, we consider the same question at advanced skill levels. In section “Coordinative AO + MI”, we discuss scenarios where observed and imagined actions are distinct, whilst in the preceeding two sections we focussed on congruent AO + MI. In section “The dual action simulation hypothesis of AO + MI”, we review the hypothesis that AO + MI engages two action simulations in parallel, and in section “AO + MI training in motor rehabilitation”, we consider motor rehabilitation as a fruitful applied field for AO + MI interventions.

Our theoretical framework is consistent with the earlier reviews by Eaves et al. (2016a) and Vogt et al. (2013). To highlight two key points here: first, we characterised *motor simulation* as an internal, real-time representation of either an observed or an imagined action, which typically involves motor cortical areas. Accordingly, AO would engage motor simulation processes *by default*, and this can assist a wide range of cognitive processes, including action recognition, action understanding, action prediction, and collaborative/joint action (Kilner, 2011; Rizzolatti & Sinigaglia, 2010). That is, we acknowledge the (well-documented) involvement of motor processes in AO, regardless of the observer’s engagement in MI (note that this does not exclude specific scenarios where AO might not engage motor simulation, e.g. Vannuscorps & Caramazza, 2016). Second, we proposed that MI (and AO + MI), whilst sharing motor simulation processes with AO, can be distinguished from pure AO in terms of the engagement of one’s own body schema, enhanced kinaesthetic processing, and by involving a greater sense of effort (James, 1890) and agency. In later sections, we expand on two further key points from our earlier reviews, namely the spectrum of AO + MI states (section“Coordinative AO + MI)”, and the potentially confounding role of MI in existing AO studies (section “The dual action simulation hypothesis of AO + MI”).

**Table:** Glossary of terms

Term	Description
Action observation (AO)	Watching human movement either via a pre-recorded video or a live demonstration
Observational learning	Structured observation of human movement over time for the purpose of acquiring and enhancing motor skills
Motor imagery (MI)	The mental representation of human movement, including its sensory and motor aspects, without physically executing the action
Motor imagery practice	Structured engagement in motor imagery over time for the purpose of acquiring and enhancing motor skills
Synchronous action observation and motor imagery (Synchronous AO + MI)	Participants observe human movement *and at the same time* imagine themselves executing either the same or a different action. When the two actions are congruent or can be coordinated, participants are normally instructed to keep the imagined action in synchrony with the observed action
Asynchronous action observation and motor imagery (Asynchronous AO and MI)	AO happens at a different time to MI, such as first observing an action and later engaging in MI, or when systematically alternating between AO and MI
Congruent AO + MI	The same action is observed and imagined simultaneously (this is the most frequently studied form of AO + MI)
Coordinative AO + MI (CoordAO + MI)	The observed action is different from the imagined action. In this case, the observer imagines an action that complements, or responds to the simultaneously observed action
Conflicting AO + MI	The observed action is different from the imagined action. In this case, the observer imagines an action that is incompatible and likely to interfere with the simultaneously observed action
Motor simulation	An internal, real-time representation of either an observed or an imagined action, which typically involves motor cortical areas
Model	Normally a human who performs a physical movement that is used as a demonstration, presented either via a pre-recorded video or in a live setting for the purpose of observation and/or imitation learning
Mixed model(s)	A human model (or models) depicting several movement attempts, which vary along dimensions such as: - Successful vs. unsuccessful attempts - Skilled vs. unskilled performers - Self vs. another person as the model - 1st vs. 3rd person visual perspectives

## AO + MI training in the early stages of skill acquisition

There are many ways in which a learner can get the initial idea of a task, including discovery learning and verbal instructions (e.g. Hodges & Franks, 2002, 2004; Lohse & Hodges, 2015). Watching a demonstration, typically of a skilled model, is however the most common starting point to convey a desired action or outcome (e.g. McCullagh & Weiss, 2002). In their ‘motor simulation and performance model’, McNeill et al. (2020) suggested AO will have a greater effect than MI in the early stages of learning and that ‘MI will have a greater relative effect on performance as expertise increases’ (p. 1). Schuster et al. (2011) also recommended MI for rehearsal and refinement of actions that exist in the performer’s motor repertoire. McNeill et al. further proposed that AO + MI will be more effective than pure AO or pure MI across skill levels. In our brief review and synthesis of the related evidence below, we concur that most studies now support these proposals.

We would further agree with McNeill et al. (2020) in predicting that physical practice should normally generate stronger performance gains when compared to AO, MI, or AO + MI, and that combinations of the latter with physical practice will be yet more effective than physical practice alone. To illustrate this point, Higuchi et al. (2012) compared imitation learning (that is: AO followed by physical execution) and a form of AO + MI training in learning guitar chords. The behavioural results indicated enhanced performance after imitation learning than following AO + MI training. In the neuroimaging data, this imitation advantage corresponded to a lack of execution-related neural resources following AO + MI compared to imitation learning. Having said this, we now turn to studies focussing exclusively on non-physical practice modalities. These non-physical modalities provide the opportunity to optimise training when physical practice is unsafe (e.g. due to injury), undesirable (e.g. to avoid overtraining) or not possible (e.g. due to time constraints or equipment availability).

### Empirical evidence

Three studies have investigated the short-term training effects (across 1 day) for AO + MI versus AO and no-practice controls. First, advantages for AO + MI training compared to a no-practice control were shown both in a visuomotor rotation task and in the related eye movements, while no such improvements occurred for two separate AO training protocols (Marshall et al., 2019). Second, in a separate study using a ball rotation task, early skill acquisition was facilitated via AO + MI training in older adults compared to a no-practice control group (Kawasaki et al., 2018). Third, as an exception to these advantages of AO + MI training, gains in a sequential reaching task were similar for the AO and AO + MI training groups in the study by Frenkel-Toledo et al. (2020). In this latter study, however, the authors conceded that their task could have provided similar opportunities for implicit sequence learning in both groups, by way of observing the sequence of the illuminated targets, rather than attending to the kinematics of the reaching arm.

Of greater relevance to the current review are four studies where AO + MI training was specifically compared to MI training. Scott et al. (2018) showed that rehearsing a Nordic hamstring drop over a 3-week period via AO + MI significantly increased peak eccentric hamstring force, while neither the pure MI group nor the no training group showed improvements. In a study by Taube et al. (2014), a 4-week non-physical balance training programme produced significant gains in both a stable and an unstable postural task. These gains were equitable across the AO + MI and MI groups, which both outperformed a no-practice control group. In a related study, 5 weeks of physical balance training in older adults produced a nonsignificant trend towards improved postural stability (Ruffieux et al., 2018). At the post-test, engaging in AO + MI of a challenging balance task significantly reduced activation in brain areas related to postural stability (which are typically overactivated in older adults), while engaging in MI only did not. Finally, Fujiwara et al. (2021) found that when learning a chopstick task with the non-dominant hand, AO + MI practice using videos of one’s own skilled performance, enhanced the perceived vividness of imagery, relative to a pure MI condition. Enhanced metabolism in prefrontal, premotor, and sensorimotor cortical regions was also identified during AO + MI, but no significant differences were found in physical task performance between AO + MI and MI. The study also showed that observing videos of another person’s hand was less effective in enhancing vividness and metabolic measures than viewing one’s own hand, suggesting that the effectiveness of AO + MI training can be modulated by the identity of the actor perceived in the video.

In four further studies, AO + MI training was compared to both pure AO and pure MI training, as well as variations of asynchronous AO + MI. Using a discrete dart-throwing task, three studies showed clear benefits for AO + MI relative to the AO and MI training groups over a 6-week period (Romano-Smith et al., 2018, 2019, 2022). Notably, these gains were equitable for synchronous AO + MI and asynchronous (i.e. alternating) AO and MI. Finally, Lin et al. (2022) found that golf putting accuracy significantly improved at the retention test following 6 weeks of synchronous AO + MI training, compared to a no-practice control group. However, two other training groups outperformed the synchronous AO + MI group in retention: an asynchronous AO and MI group, and a hybrid AO + MI group, comprising asynchronous AO and MI early in learning, followed by synchronous AO + MI later in learning. In the studies of asynchronous AO and MI, however, the instructions did not aim to prevent spontaneous MI during the AO segments. If this had occurred, the design would amount to a more intense schedule alternating between AO + MI and MI, rather than plainly alternating between AO and MI.

Overall, the evidence reviewed here is relatively consistent and largely supports McNeill et al.’s (2020) proposal for an advantage of AO + MI training in early stages of practice, compared to either AO or MI alone. Given the relatively small number of training studies available, however, a systematic approach to the study of AO + MI effects is now warranted. A challenge for future studies contrasting AO + MI practice with pure MI practice will be to separate the provision of additional *information* about the required task, as conveyed in the AO component of AO + MI, from genuine practice effects. Careful consideration should also be given to control for training volume (particularly when comparing synchronous AO + MI to asynchronous AO and MI) and to consider action type (e.g. movement form vs. outcome-driven tasks), and the potential effects of task complexity.

### AO + MI effects in early motor learning: explanations and considerations

Assuming this general trend for advantages of AO + MI over both the pure AO and pure MI protocols continues to be replicated in future research, the critical question is how can these gains be explained? Here, we outline two conceivable accounts, additive and super-additive, and we expand on specific scenarios for the latter. A first, parsimonious explanation would assume a plainly additive effect of combining the component AO and MI processes. That is, the internally generated motor simulation (MI) and the externally induced visuomotor representation of the same action (AO) both increase and expedite motor processing independently, and this facilitates motor learning. This explanation would indeed sufficiently account for the results reviewed above.

A second and complementary explanation refers to super-additive effects of combining AO and MI, where *interactions* between synchronous AO and MI processes offer unique solutions to the limitations inherent in separate AO and MI training protocols. Compared to pure MI protocols, these putative interactions would strengthen the real-time motor simulation. A defining and potentially limiting characteristic of pure MI is the absence of any external reference. Indeed, the large body of research into MI suffers from a degree of uncertainty over what participants actually imagine and over its precise timing, despite efforts to run manipulation checks (Ladda et al., 2021; Schuster et al., 2011). By contrast, AO + MI instructs close attention to the observed target action. Through AO, the essential spatio-temporal characteristics are continuously specified, which allow refinement and updating of the internal simulation in real time. For researchers, AO + MI has an advantage in terms of increased control over core features of the motor simulation process. Indeed, recent neuroimaging work has confirmed that specific movement phases can be decoded more accurately from the data obtained during AO + MI than from data obtained during MI or AO alone (Kaneko et al., 2018, 2021; Suzuki et al., 2021; Yokoyama et al., 2021). In the context of Bach et al.’s (2022) proposal for effect-based MI processes, one could also argue that the real-time character of AO + MI likely biases the practice to include lower-level motor processing.

Regarding AO + MI-related practice effects, possible super-additive effects can be studied by contrasting alternating AO and MI against synchronous AO + MI. The related evidence is at present rather scarce and, where available, not indicative of advantages for the latter (Lin et al., 2022; Romano-Smith et al., 2018, 2019, 2022,  but see Sun et al., 2016, for advantages of synchronous AO + MI over asynchronous AO and MI in stroke rehabilitation). Thus, further research on the matter is clearly warranted. There are at least two related challenges facing research in this area. First, it is difficult to assess the accuracy of synchronisation between concurrent AO and MI processes. One solution would be to instruct, along with AO, ‘dynamic’ MI (Guillot et al., 2021), where participants are asked to make small movements expressing their imagery content, which can be tracked and compared to the observed time course. Second, because of the possible confound of spontaneous MI during AO phases within alternating AO and MI schedules, control conditions are needed to minimise carry-over effects.

Interaction effects during AO + MI might also refer to possible differences in the specificity of the AO component relative to the MI component. For example, Wright et al. (2018a) showed that when visually attending to different body parts in the display, gaze behaviour moderated corticospinal excitability. Two separate studies demonstrated that observing particular body parts activated cortico-motor regions corresponding to the same body part in the observer’s brain (D’Innocenzo et al., 2017; Puglisi et al., 2017). Although the evidence for effector specificity after AO have been mixed (e.g. Bird & Heyes, 2005; Williams & Gribble, 2012), AO might facilitate better effector-specific encoding of motor programs, that is, mapping specific movement parameters to a particular body part, compared to MI. Research has indeed shown that MI can promote effector *independent* encoding, relating to acquisition of global movement features, like rhythmical timing and spatial consistency (Kraeutner et al., 2017, 2020). The supplement of AO to MI might, therefore, promote a unique hybrid modality for motor learning that involves parallel encoding of both the effector dependent (AO) and effector independent (MI) action features.

AO and MI might also make different contributions to the development of *mental representations of action*. According to Kim et al. (2020), who compared asynchronous AO + MI with separate AO and MI practice conditions, these are cognitive representations comprising a series of body postures and associated sensory consequences, which are related to successful motor execution (see also Frank et al., in press). Wright et al. (2018b) suggested AO + MI practice might, therefore, enhance mental representations in terms of the AO component supporting sequencing and timing, while the MI component might enhance the associated sensory consequences, leading to improvements in motor learning.

Finally, it is important to note that interactions between concurrent tasks can also produce interference. For example, there is evidence of interference between verbal and motor tasks. In experimental paradigms where participants were required to attribute a meaning to verbs expressing concrete, effector-specific actions and provide their choice by means of hand or foot motor responses, participants gave slower hand motor responses when they processed hand-related verbs as compared to foot-related verbs (Buccino et al., 2005; de Vega et al., 2013; Garofalo et al., 2022; Klepp et al., 2015; Sato et al, 2008), specifically within 200 ms from stimulus presentation. Given that the verbal and motor tasks used involve partially the same neural structures (Hardwick et al., 2018), these interference effects were interpreted to reflect a cost of concurrent engagement of the same neural resources in the motor system (for review see Buccino et al., 2016). Extrapolating from these results to AO + MI, one might expect similar interference between the AO and MI components. Although the evidence thus far mostly points to benefits from combining AO and MI, one should be aware of potential task interference effects under certain conditions, and that the timing between the tasks might be critical. Whilst these considerations primarily apply to congruent AO + MI, interference effects might also emerge from heterogeneous and incompatible contents of the AO and MI components (see ‘Conflicting AO + MI’ in Vogt et al., 2013).

We hope this synopsis of possible explanations of AO + MI effects will motivate future research. In the next sections we consider several scenarios that have received attention in the observation and motor learning literature but have not yet been investigated in the context of AO + MI training. We also consider variables that are likely to moderate the effectiveness of supplementing MI practice with concurrent AO.

### Observing errors and mixed-skill models in observational learning practice and AO + MI

An important question in relation to observational learning is what type of model to provide? There is extensive literature related to watching successes versus errors, and viewing learning models or watching one-self perform (McCullagh & Weiss, 2001; McCullagh et al., 2012). One prominent finding is that seeing a mixture of skilled and less-skilled models has been shown to be a beneficial learning method (e.g. Andrieux & Proteau, 2013, 2014; Rohbanfard & Proteau, 2011). These mixed-skill models, showing different types of performance are thought to help the learner detect errors in their own actions (by seeing the desired action), and correct these errors (from seeing unsuccessful attempts improve over time). There is also evidence that novices overestimate their accuracy during imagery in comparison to actual execution (e.g. Rieger et al., 2011; Dahm & Rieger, 2019, see Rieger et al., in press). Seeing a mixture of models and performance outcomes during AO + MI may, therefore, promote a more veridical imagery process better matched to actual success.

The observation of errors, rather than flawless performance, has been identified as a beneficial aspect of observational learning at early stages of training in sport (Hodges, 2017), surgery (LeBel et al., 2018), and even fundamental motor skills (Brown et al., 2010). Little evidence exists showing how gains that are made from observing the mistakes of other people can be facilitated (or interfered with) by synchronous imagery. In the only available AO + MI study on this topic, watching an errorful model was a beneficial supplement to MI for elderly participants who learned a ball rotation task (Kawasaki et al., 2018). AO + MI of a slow model that included an error in their performance produced significant learning gains, compared to a group who undertook AO + MI of a skilled performer and compared to a no-practice control group.

In the AO literature, there is also evidence that unanticipated outcomes can lead to an automatic compensatory-type process after watching (Ikegami et al., 2018). For example, seeing someone aim for and then miss a target can result in small unintended compensatory behaviours, or ‘after-effects’ in the observer’s subsequent action execution (Ikegami & Ganesh, 2014; Ikegami et al., 2018; Ronchi et al., 2011). Even in simple motor tasks, the observation of errors might lead to performance improvements by signalling key information for an actor’s future performance. For example, Buckingham et al. (2014) presented evidence showing that observation of errors led to improvements in sensorimotor prediction. Observing a video of another individual lifting a heavy-looking object with too much force led to the participant reducing the force they would subsequently use to lift the object. Measurements of corticospinal excitability indicated that this behavioural improvement was driven by automatic responses to inherent cues about object mass within the video. That is, the response to heavy-looking objects was cancelled out by the response to light-looking kinematics within the observed overestimation across trials. Whether the apparently useful information gained from errors in this simple context extends to more complex tasks is an open question. But this scenario does highlight a possible role for imagery in a more multisensory context. For example, one can readily imagine how it feels to overestimate the weight of an object when lifting it—the rapid uncontrolled accelerations and sharp deceleration at an uncomfortable height is common enough and is experienced through tactile (Johansson & Flanagan, 2009) and visual channels (Buckingham et al., 2011). It is plausible that explicitly asking participants to imagine the sensory consequences of commonly experienced ‘everyday’ errors (e.g. what an overestimation might feel like) might further enhance the effect of this error-based observational learning.

The question of relevance to MI is how unsuccessful models and the observed behavioural effects might interact with on-going MI. It may be that these compensatory effects are cancelled out by imagining successful outcomes, preventing unintended errors in experienced performers. To date, however, there has not been research on the interactive effects of AO and MI when individuals are watching errors.

### Variability of practice in AO + MI training

The variability of practice hypothesis for motor learning offers an intuitive framework for exploring and contextualising a variety of novel AO + MI arrangements, including the observation of errors as discussed above. In Schmidt’s (1975) influential theory of generalised motor programs, one proposal was that an increased range of parameter experiences should enhance motor learning. For example, shooting a basketball at a hoop from various distances results in variable practice of skill-related parameters. A large body of evidence supports the idea that this kind of variable practice is more effective for learning and transfer than constant practice (i.e. repeatedly shooting from a single distance; Wulf et al., 2010). AO + MI training offers an opportunity to explore if these well-established principles of physical practice also hold true in mental practice. Regarding pure AO, there is already evidence that variable observation conditions, such as observing different angular positions in an arm aiming task, enhance learning in comparison to constant observation conditions (Bird & Rikli, 1983).

If we consider the case of variable AO + MI, the video recordings of a javelin throw, for example, could vary across trials in one or several dimensions (e.g. the angle of release, posture, speed, footwork, or movement amplitudes), and learners could be asked to engage in congruent AO + MI, that is, they would align their MI to the specific parameter set of the observed variable javelin throws. In a more adventurous version of variable AO + MI practice, imagined and observed action could be incongruent. For example, participants could be asked to vary their MI regarding a certain dimension across trials whilst observing a constant display, or they could engage in consistent MI of the same action across trials, while at the same time parameters in the visual display are varied.

The gains from variable physical practice are typically larger in children than adults, since parameter specification is in a formative stage at younger ages (Wulf et al., 2010). Given the evidence that MI enhances motor learning in children (Behrendt et al., 2021), and given the efficacy of AO + MI training in children both with and without motor impairment (Marshall et al., 2020; Scott et al., 2019, 2020, 2021), children are the ideal model for assessing the effectiveness of the above scenarios of variable AO + MI training.

### Using AO as surrogate visual feedback in MI

When we act, we see the visual consequences of our actions, but we do not see what our actions look like from multiple perspectives, unless we perform in front of a mirror. There is research showing that online visual feedback of action components that cannot be seen, or are difficult to see, can supplement observational learning from a model. The benefits of combining demonstrations with visual feedback were shown both when the video feedback was from the same external (3rd person) perspective as the demonstration (Carroll & Bandura, 1982), and when displayed from the internal (1st person) perspective of the performer (Hodges & Franks, 2002). Observing difficult-to-perceive action components, in the sense of surrogate visual feedback, may, therefore, also be one factor explaining the facilitatory effects of AO + MI training. The study by Fujiwara et al., (2021, see section “Empirical evidence”) nicely exemplifies this, as the videos used for AO + MI training with the non-dominant hand were flipped versions of the participants’ earlier performance with the dominant hand, so that they resembled visual feedback as closely as possible.

There is also research directed to the question of what perspective is best for observing or imagining (see Wright et al., 2021). For example, beginners often prefer *external* MI and AO perspectives, where they see or imagine looking at someone else performing an action, over *internal* perspectives where the view is of the individual who is acting on an object (Hardy & Callow, 1999; Montuori et al., 2018). The external perspectives (either AO or MI) are thought to guide performance and learning, facilitating change, whereas the internal perspectives activate already acquired action representations, only if and when they exist (Montuori et al., 2018). This could mean that transitioning from external to internal perspectives with practice would be maximally beneficial for an AO + MI learning protocol, but to date this has not been researched.

### Factors moderating the effectiveness of AO + MI training

Several factors are likely to modulate the effect of AO + MI interventions at the outset of learning. Imagery ability plays a mediating role in generating and controlling the vividness of MI experiences (Cumming & Eaves, 2018). If individuals have poor imagery ability, we might expect that supplementing MI with AO would be beneficial for practice. To date, however, there is no evidence attesting to this specific benefit of AO + MI for poor imagers. In a comparison between expert golfers classified into either ‘good’ or ‘poor’ imagery groups, there was no difference in putting performance following an AO + MI intervention (McNeill et al., 2020). However, this was a study of motor performance rather than learning, and the so called ‘poor’ imagers did not score at the low end of the imagery ability scale (Robin & Blandin, 2021).

Age may be an important moderating factor, too, given that balance training via AO + MI instructions has been effective in younger (Mouthon et al., 2015) but not older adults (Mouthon et al., 2016). Benefits for AO + MI instructions have also been shown in children (7–11 years) who performed instantaneous imitation of familiar actions (Scott et al., 2020), whereby AO + MI effects were more pronounced in children who had an increased proficiency in fundamental movement skills. There are likely other individual difference factors, such as attentional capacity and preferences for imagery perspective, that might influence both the extent to which and the timing of when learners can move from pure AO or MI to AO + MI trainings.

In summary, we have highlighted the important role of AO-related processes in early skill acquisition and the benefits of AO + MI over either pure AO or pure MI in this context. We have also made practical suggestions for supplementing MI with AO, and we have described when and why these may be effective for enhancing the early stages of motor learning.

## AO + MI training at advanced skill levels

What might be the advantages and disadvantages of AO + MI, compared to pure MI, at later stages of practice? In the absence of related research, we confine our discussion to some principal considerations and suggestions. At these later stages, a representation of the action is firmly established and can be activated internally via MI alone (Hetu et al., 2013). There is good reason to believe that individuals with physical action experiences will differ in their response to MI, and to combined AO + MI, due to their ability to engage the motor system through simulation-type mechanisms. For example, activation of the Action Observation Network (AON; including dorsolateral premotor cortex, inferior parietal lobe, and superior temporal sulcus) via AO is highly influenced by prior physical experiences (e.g. Calvo-Merino et al., 2005, 2006; Cross et al., 2009; Higuchi et al., 2012; Sakreida et al., 2018; Vogt et al., 2007, for reviews see Karlinsky et al., 2017; Ramsey et al., 2021). Outcome predictions also differ in their mechanisms and accuracy depending on the experiences of the observer. People with physical experiences, whether experts in basketball (e.g. Abreu et al., 2012; Aglioti et al., 2008) or individuals trained in dart throwing (e.g. Ikegami & Ganesh, 2014; Mulligan et al., 2016a), show evidence consistent with increased activation of their motor system during observation for prediction. This has been inferred through transcranial magnetic stimulation (TMS) and resulting motor evoked potentials (MEPs), as well as through secondary motor task interference specific to the effectors involved in the action. Whilst motor experience normally facilitates processes of AO, MI, and AO + MI, it is worth mentioning that recognition and prediction tasks can also rely on non-motor mechanisms (e.g. Mulligan et al., 2016b; Vannuscorps & Caramazza, 2016).

The propensity of motor experts to activate brain areas that overlap with execution (i.e. the AON) when watching, is thought to reflect their ability to engage in simulation/motor resonance. MI can also involve some of these same AON areas, including premotor cortex and inferior parietal lobe (e.g. Lebon et al., 2018; Solomon et al., 2021; Zabicki et al., 2019). MI has additionally been linked to activations in the cerebellum and primary motor cortex (Hardwick et al., 2018; Hetu et al., 2013). In section “The dual action simulation hypothesis of AO + MI”, we expand further on the neural separability of AO and MI and their potential to serve different functions. Indeed, combining AO with MI activates more diverse brain areas (Ruffieux et al., 2018; Taube et al., 2015), which could impact training effectiveness in skilled athletes.

For congruent AO + MI, where observed and imagined actions coincide, comprehensive recommendations for AO + MI interventions in sport have been given (Wright et al., 2021). These recommendations include focussing the learner’s attention, through edited videos, on specific aspects of the modelled performance, such as on individual body parts or performance-critical time points. A possible issue of AO + MI for athletes, particularly immediately before performing in a competition, is that observing another athlete could derail, rather than stabilise one’s own motor representation (Ikegami & Ganesh, 2014). Whilst pure MI avoids this issue and is typically used pre-competition, AO + MI of one’s own video-recorded best performance (i.e. self-modelling) might provide an interesting alternative here. To date, there has only been one study where ‘self’ versus ‘other’ videos have been compared in an AO + MI intervention with skilled golfers (McNeill et al., 2021). While club-path kinematics showed stronger improvement in the ‘self’ group, putting accuracy did not differ between the ‘self’ and ‘other’ groups at the post-test.

AO combined with MI, rather than MI alone, could also facilitate improvements in experienced performers by way of seeing one-self in a future, yet unattained state (Wright et al., 2021). For example, Aoyama et al. (2020) demonstrated that, after reaching a plateau in their learning of a ball rotation task, participants made significant improvements using AO + MI of a moderately better performance compared to a control and two other AO + MI conditions, which displayed either the learners’ current or a significantly better than current performance. Frank et al. (2022) found a similar result with novice participants practising squats. In their AO + MI protocols, participants observed an avatar depicting themselves performing either one of their own previously executed squats (Me-Novice), or an avatar of themselves that had been edited in virtual reality to perform a skilled squat (Me-Skilled). Advantages were found for the Me-Skilled group in movement kinematics and cognitive representation structure. By showing learners a just out-of-reach, future state (or unachieved outcome), they may engage in more vivid MI practice of that skill, facilitating future attempts. This approach is compatible with applied frameworks for technique refinement via mental practice (see Carson & Collins, 2016) and mental practice using layered stimulus response training (see Cumming & Eaves, 2018), which target performance enhancement in athletes.

For experienced individuals, the internal (1st person), rather than the external (3rd person), perspective of imagery is thought to serve a revision rather than learning role, for an already internalised set of movements (Montuori et al., 2018). This hypothesis was based on data showing that experienced Pilates performers matched their actual execution times better during internal- rather than external-visual imagery, whereas the opposite was shown for beginners (external was better matched, Montuori et al., 2018). Better time matching is thought to provide evidence of an enhanced ability to internally simulate (Decety et al., 1989; see also O’Shea & Moran, 2017). As such, if an experienced performer’s aim is to solidify their existing movement technique, AO of an internal perspective would be a preferable complement to their internal MI perspective. If the requirement is to signal a technique change, supplementing internal MI with external AO could be advantageous for experienced performers.

## Coordinative AO + MI

Most research on AO + MI, as well as our above considerations, has so far focussed on *congruent AO* + *MI*, where essentially the same action is observed and imagined. In their original position paper, Vogt et al. (2013) proposed a wider spectrum of AO + MI states, spanning from congruent AO + MI to *coordinative AO* + *MI*, where the observer imagines performing an action that complements, or responds to the observed action, to *conflicting AO* + *MI*, where observed and imagined actions are incompatible and likely interfere with each other. Here, we highlight coordinative AO + MI (coordAO + MI) as a further, fascinating scenario for study. CoordAO + MI can be regarded as a special case of joint action (Sebanz & Knoblich, 2021; van der Wel et al., 2021), because the observer imagines responding to, or performing with the actions of another person. In coordAO + MI, however, one’s own action is not executed, as it would be in joint action. Vogt et al. (2013) illustrated coordAO + MI with an example from combat sports: “I might watch a video recording of a future opponent whilst simultaneously imagining myself performing specific technical attacks or defence movements against that opponent” (p. 1). For any sports where fast, seamless responses to an interacting partner or opponent are crucial, coordAO + MI interventions are an attractive adjunct to (joint) physical practice. To give a further example, one of us (SV) has recently developed a related intervention where elite athletes watch a number of video-recorded fencing attacks in random order and imagine their own appropriate defence or counter-attack responses. Note that it is also conceivable to employ virtual reality technology for such coordAO + MI interventions (c.f., Lindsay et al., 2022).

Situations where interpersonal coordination is sustained, such as couples’ dancing or ensemble music, are also likely scenarios where coordAO + MI training would be beneficial (see Ladda et al., 2020). An advantage of coordAO + MI, compared to ‘the real thing’, is that learners can flexibly focus on a single observed body part at a time. In addition, learners can focus on specific aspects of their own motor image, whilst joint action normally requires a fully articulated action. Since the observer can see the action of a partner or opponent unfold in real time, imagery training is both dynamic and predictive, where the spatio-temporal couplings are maintained. There may also be a safety or efficiency benefit associated with coordAO + MI, where in sports such as baseball or cricket, hitters or batters only need to imagine responding to a fastpitch or bowl behind a safety barrier or in response to video. Such coordAO + MI training may benefit predictive capacity and could be coupled with sport-based perceptual training methods, where videos are edited to occlude outcomes (such as a serve in tennis, e.g. Smeeton al., 2005) and athletes imagine responding, rather than the typical response of providing a verbal prediction of outcome.

## The dual action simulation hypothesis of AO + MI

Understanding the neural foundations of AO + MI is both a fascinating and under-researched endeavour. We first briefly revisit research where AO and MI were studied separately. The most encompassing meta-analysis to date of neuroimaging studies of AO, MI, and execution (Hardwick et al., 2018) confirmed that both AO and MI include processing in brain regions that are traditionally classed as ‘motor’, which concurs with Jeannerod’s (2001) proposal that both AO and MI involve motor simulation processes. Specifically, overlap between AO and MI was found in lateral and medial premotor and rostral parietal regions (see ‘AO network’ in section “AO + MI training at advanced skill levels”). Importantly, the volume comparisons of Hardwick et al. also indicated that AO and MI each engage large sets of voxels that were *not* shared. This was the case for 61% of all activated voxels for AO (notably in occipito-temporal regions), and for 47% of the voxels for MI (including the left dorsolateral prefrontal cortex and regions of the cerebellum and basal ganglia). It thus appears that the story of a ‘wide activation overlap between AO and MI’, as endlessly repeated even in the most recent literature, ignores that AO and MI also involve unique neural substrates.

The activation overlap between AO and MI is also likely to have been overestimated when considering that the instructions used in studies on putative pure AO rarely aimed to prevent spontaneous MI during AO, that is, AO + MI (Vogt et al., 2013). Whilst it is ultimately impossible to establish retrospectively the amount of such a contamination of AO by spontaneous MI, Thorne (2020) rated the instructions of the 498 neuroimaging studies on AO in Hardwick et al.’s (2018) meta-analysis, and found that approximately 200 studies had instructions that likely encouraged concurrent MI. This previously unnoticed confound would thus inflate related estimates of the neural overlap between putative pure AO and MI (see also discussion in Hardwick et al., 2018). Thorne (2020) also conducted separate ALE meta-analyses of the AO studies where spontaneous MI was either rated ‘likely’ or ‘unlikely’. The first key finding was that both subsets engaged the fronto-parietal ‘mirror’ regions to a similar extent. This result corroborates the notion that AO engages motor simulation processes by default, regardless of any MI involvement. The second key finding was that the anterior insula, a region involved in somatosensory processing (Craig, 2009), was only found engaged in those AO studies where MI was ‘likely’ involved. This finding is consistent with our proposal that during AO + MI (here, AO conditions that indirectly encouraged AO + MI), one’s own body schema is engaged more strongly than during pure AO.

Let us now turn to AO + MI proper. In our discussion of the behavioural effects of AO + MI training, we implicitly assumed that the neurocognitive component processes of pure AO and pure MI remain largely intact during AO + MI, and that they either operate independently of each other (additive explanation) or that they coexist and interact in some form. The core proposal that AO + MI might involve two parallel, separable motor simulation processes, has been termed “*dual action simulation*” (DAS, Eaves et al., 2016a; Vogt et al., 2013). This proposal was based on behavioural work (Eaves et al., 2012, 2014, 2016b) and on the wider framework of biased competition (Cisek & Kalaska, 2010). In what ways has neuroscientific research informed the DAS hypothesis? The available neuroimaging studies on AO + MI generally indicate more robust activation of motor-related brain regions, relative to pure AO or pure MI conditions, as demonstrated using functional magnetic resonance imaging (fMRI, Macuga & Frey, 2012; Nedelko et al., 2012; Taube et al., 2015; Villiger et al., 2013), electroencephalography (EEG, Berends et al., 2013; Eaves et al., 2016b; Neuper et al., 2009), TMS (Sakamoto et al., 2009; Tsukazaki et al., 2012; Wright et al., 2014, 2018b) and functional near-infrared spectroscopy (fNIRS, Holper et al., 2010, 2012). While the extent of activations during AO + MI could be coarsely described as a superset of the activated regions during pure AO and pure MI, these results can, at best, only be taken as tentative support for the DAS hypothesis: We know that AO and MI partially overlap, and also the documented differences between the two (Hardwick et al., 2018) are not unambiguous markers for the presence of each (AO or MI) component. Rather, the core assumption of the DAS hypothesis is that the two simulations are carried out in the very same brain region(s), as we discuss now.

To date, only two studies have addressed the DAS hypothesis directly. In both studies, TMS of the primary motor cortex was used to probe corticospinal excitability of the hand muscles. Meers et al. (2020) asked participants to fixate on a small centred marker and to observe a rhythmical movement of, e.g. the index finger whilst imagining moving their little finger in synchrony, which is a form of coordAO + MI. Results showed that corticospinal excitability exclusively reflected the finger engaged in MI, whilst the AO component did not exceed baseline levels. These findings do not support the DAS hypothesis, which would predict that AO and MI components produce comparable facilitatory effects. It appears that in this experimental paradigm the observed action was primarily used as an external-visual scaffolding for MI (‘visual guidance hypothesis’, ibid.), rather than activating a separate motor representation. In the second study, a similar design was used but participants’ eye movements were also tracked (Bruton et al., 2020). The main analysis broadly replicated the results of Meers et al. (2020), whereby the AO component did not significantly facilitate MEPs above baseline in the index finger muscle during coordAO + MI. However, when the data were separately analysed based on eye fixations on the index finger, the AO component of the coordAO + MI condition did facilitate MEPs above baseline in the index finger muscle. Since MI of the little finger also facilitated MEPs in the corresponding muscle, these data support the DAS hypothesis. Nevertheless, regardless of the type of control condition or analysis, the MI component still produced the dominant effect, in line with the results of Meers et al. (2020). Thus, further research, ideally including regions upstream of primary motor cortex, would be required before firm conclusions regarding the DAS hypothesis can be reached here.

The hypothesis of multiple motor representations is not limited to AO + MI scenarios: supporting evidence for the brain’s capacity to simulate more than one action at a time has come from studies on joint action (e.g. Ménoret et al., 2015) and from several studies on observing multiple agents (e.g. Cracco et al., 2019). Through Multi-Voxel Pattern Analysis (MVPA) of fMRI data, Cracco et al. (2019) decoded two different concurrently observed hand postures in premotor and posterior parietal cortices. MVPA methodology has also been used to successfully decode different imagined actions from fMRI signals in frontal and parietal regions (Pilgramm et al., 2016; Zabicki et al., 2017). Based on these findings, an application of MVPA methodology for testing the DAS hypothesis of AO + MI would, therefore, appear both feasible and highly desirable. As in the above TMS studies, participants would need to engage simultaneously in AO of action X and in MI of action Y, and MVPA could be employed to decode each of the two actions in the same fMRI signal, with premotor and inferior parietal cortex as the main target regions. It is also conceivable that in AO + MI tasks, cortical processing becomes biased towards a stronger *segregation* (i.e. less overlap) of activations compared to pure AO and pure MI tasks, for example, occipito-temporal regions for the observed action and fronto-parietal regions for the imagined action. Such an outcome would support an alternative version of the DAS hypothesis where the two simulations are in part carried out in different brain regions.

## AO + MI training in motor rehabilitation

In terms of applications, in the previous sections, we mainly had the acquisition of sport skills in mind. We now shift the focus on to employing MI, AO, and AO + MI in the rehabilitation of motor impairment in different neurological and non-neurological diseases. While there is a promising approach for MI in patients with Parkinson’s disease (e.g. Tamir et al., 2007; see Abraham et al., 2021), mixed results have been obtained for MI practice in the recovery of stroke patients (see Braun et al., 2013; Butler & Page, 2006; Emerson et al., 2018; Liu et al, 2004; Machado et al., 2015; Zimmermann-Schlatter et al., 2008). Some studies have suggested that patients with damage to specific brain structures, including the parietal and frontal lobes, lose the capacity to imagine motorically (e.g. McInnes et al., 2016; see Braun et al., 2013). In addition, where MI is spared, it likely draws on executive cognitive resources to a greater extent than motor execution (Glover et al., 2020). The use of MI as a rehabilitative tool may, therefore, have limited value for certain patient groups.

Action Observation Treatment (AOT, Buccino, 2014) has been proposed as an alternative and more widely applicable rehabilitation tool, exploiting the physiological evidence that AO, as well as MI, recruits to some extent the same neural structures as physical execution (Hardwick et al., 2018; Jeannerod, 2001; Rizzolatti et al., 2021). During a typical AOT rehabilitation session, patients train a single action of daily living, such as having an espresso, or washing their hands. During the *observation phase*, the patient sits in front of a computer screen and carefully observes a video clip depicting the daily action, and in the subsequent *execution phase*, they physically perform the observed action with objects provided. AOT has been widely used in the recovery of motor functions in stroke patients (e.g. Ertelt et al., 2007, see Rizzolatti et al., 2021; Ryan et al., 2021), patients with Parkinson’s disease (Buccino et al., 2011; Pelosin et al., 2010), children with cerebral palsy (Buccino et al., 2012, 2018; Sgandurra et al., 2013) and patients undergoing orthopaedic surgery for hip/ankle replacement (Bellelli et al., 2010). At least in children, AOT has also been used successfully in a telerehabilitation setting (Molinaro et al., 2020).

AO + MI treatment (or training) represents a meaningful combination of the two above approaches to motor rehabilitation, and the available behavioural and neurophysiological findings speak to the likely benefits of this combination. Compared to AOT, AO + MI treatment essentially involves the flexible integration of MI processes in the training, either synchronously or in alternation with the observation phase. In stroke recovery, Sun et al. (2016) found clinically relevant improvements both in measures of motor performance and in cortico-motor involvement (assessed via EEG) in 10 chronic stroke survivors who undertook synchronous AO + MI treatment alongside physical rehabilitation. Similar gains were not achieved in a separate group who received asynchronous AO and MI treatment alongside physical rehabilitation. In a randomized controlled trial, Choi et al. (2022) also showed improvements for AO + MI compared to AO therapy in stroke survivors, in the both the Fugl-Meyer Assessment (FMA) of upper-limb function, and using TMS, which demonstrated increased corticospinal excitability over 5 weeks for AO + MI but not AO therapy. Robinson-Bert and Woods (2022) also found improvements in upper-limb motor recovery (FMA scores) for AO + MI. practice, but only in sub-acute stroke patients with increased commitment to the intervention. In Parkinson’s disease, research indicates facilitatory effects of AO + MI instructions both in the objective and subjective measures of performance of manual actions, compared to AO (Bek et al., 2018, 2021; see Caligiore et al., 2017). Gains in fundamental movement skills have also been achieved by supplementing MI with AO in children with developmental coordination disorder, compared to either pure AO or pure MI interventions (Marshall et al., 2020; Scott et al., 2019, 2020; Steenbergen et al., 2020; see Scott et al., 2021). AO + MI instructions have further been used to successfully counteract a pain-induced reduction in cortico-motor excitability (Larsen et al., 2019; see Suso‐Martí et al., 2020), while AO + MI treatment has also been recommended for reducing adverse effects on the motor system following immobilisation-induced hypoactivity (Monany et al., 2022). Positive results of AO + MI treatment have additionally been obtained in muscular rehabilitation following hip replacement (Marusic et al., 2018; for related studies on strength training with healthy participants, see Shimada et al., 2019; di Rienzo et al., 2019). Finally, there is evidence of the potential benefits of using AO + MI instructions in brain–computer interface paradigms (e.g. Neuper et al., 2009), for example, incorporating proprioceptive neurofeedback (Ono et al., 2018).

Encouragingly, these findings advocate for an integrated AO + MI treatment, tailored to the specific clinical populations, and no study to date has identified a clear-cut contraindication of AO + MI treatment compared to AOT. Future research should now focus on potential issues to help optimise AO + MI applications: First, in the early stages of rehabilitation it is likely that not all patients will have the capacity for MI or for synchronised AO + MI, thus strengthening MI ability would be an appropriate goal. Second, as mentioned above, for certain patient groups with motor impairment, the capacity for MI and AO + MI might be permanently compromised, and AOT would then be the preferred option. Third, synchronous AO + MI tasks likely require additional neurocognitive effort. In two related studies with healthy participants, cortical activation in prefrontal regions significantly increased during synchronous AO + MI tasks compared to either pure AO or pure MI (Eaves et al., 2016b; Emerson et al., 2022, see also Fujiwara et al., 2021). This suggests that additional operations of cognitive control and monitoring preside over the component AO- and MI processes. Despite this, participants subjectively report that synchronous AO + MI feels less effortful than pure MI when generating a motor simulation (e.g. Bruton et al., 2020). Nevertheless, future studies assessing the most suitable ways of combining AO and MI should bear in mind that synchronous AO + MI treatment might be overly demanding for certain patient groups or stages of rehabilitation (Emerson et al., 2018). In this case, asynchronous AO and MI, with alternating phases of AO, MI, and execution, would provide a potentially more suitable alternative.

Applications of AO + MI training are also conceivable outside sports and neurorehabilitation. Let us conclude with just one example, surgical education. Green et al. (2019) recently reviewed the benefits of video observation in this field (see also Harris et al., 2018; Rizzolatti et al., 2021), which has shown positive effects with novice learners in a range of disciplines from arthroscopy (LeBel et al., 2018) to robot-assisted surgery (Harris et al., 2017). Goble et al. (2021) and Snelgrove and Gabbott (2020) came to similar, positive conclusions in their reviews of MI in surgical training. We can only hope that history repeats itself, in that AO and MI, which a decade ago were studied and applied in isolation of each other, will be considered from an integrated perspective in future research on surgical education.

## Conclusions

Our aim in this paper was to explore the benefits and issues that can be expected from adding AO to MI in a synchronous manner, with a focus on motor skill acquisition. We have highlighted the important role of AO-related processes in early skill acquisition and reviewed the rapidly expanding literature on the practice effects of AO + MI, compared to either AO or MI training. We have made practical suggestions for supplementing MI with AO, and we have described when and why these may be effective in motor learning. We have also described how coordinative AO + MI offers opportunities for novel training applications in both experienced and novice performers, and we reviewed the dual action simulation hypothesis on the neurocognitive mechanisms of AO + MI. Finally, we considered AO + MI effects on rehabilitation across several populations and offer considerations for optimising AO + MI applications in this context. 

Throughout this paper, our focus has been deliberately set on synchronous AO + MI. While the instruction to synchronise the imagined and observed actions normally supports and enhances MI practice, there could still be settings where this synchronisation requirement might be overly constraining for the learner. In such cases, asynchronous forms of AO + MI should be considered, along with our proposal for variable AO + MI training. Overall, we believe this exploratory review makes a convincing case for the flexible use of AO + MI practice in a variety of scenarios, either on its own or in combination with physical practice, as an alternative to mental trainings that rely on either AO or MI alone. It is clear much empirical work remains to be done towards an interdisciplinary and integrated approach, aiming to understand the interplay between different forms of action representations and the implications for applied disciplines.

## Data Availability

Data sharing not applicable to this article as no datasets were generated or analysed during the current study.

## Footnotes

1 Note that the present paper takes a line of enquiry that is complementary to our earlier reviews (Eaves et al., 2016a; Vogt et al., 2013). Our approach there was grounded in the large research base on AO and its neural underpinnings, where we considered possible contributions of MI to AO. We addressed the (implicit) question ‘Why bother with MI’, or with AO + MI, when AO seems to produce similar neurocognitive effects with better stimulus control’? In the present paper, we pursue the inverse question ‘Why bother with AO?’ and hope to show that focussing on the contributions of *AO to MI* is an equally worthwhile and inspiring undertaking.
